# Rise and Demise of Bioinformatics? Promise and Progress

**DOI:** 10.1371/journal.pcbi.1002487

**Published:** 2012-04-26

**Authors:** Christos A. Ouzounis

**Affiliations:** 1 Institute of Agrobiotechnology, Centre for Research & Technology Hellas (CERTH), Thessaloniki, Greece; 2 Donnelly Centre for Cellular & Biomolecular Research, University of Toronto, Toronto, Ontario, Canada; University of California San Diego, United States of America

## Abstract

The field of bioinformatics and computational biology has gone through a number of transformations during the past 15 years, establishing itself as a key component of new biology. This spectacular growth has been challenged by a number of disruptive changes in science and technology. Despite the apparent fatigue of the linguistic use of the term itself, bioinformatics has grown perhaps to a point beyond recognition. We explore both historical aspects and future trends and argue that as the field expands, key questions remain unanswered and acquire new meaning while at the same time the range of applications is widening to cover an ever increasing number of biological disciplines. These trends appear to be pointing to a redefinition of certain objectives, milestones, and possibly the field itself.

This is an “Editors' Outlook” article for *PLoS Computational Biology*.

## In Lieu of an Introduction

After considerable deliberation and multiple discussions with colleagues over the last couple of years, and having written several retrospective assessments, I would like to touch upon yet another historical aspect of the field of computational biology [1]. The intention here is to explore the rise and demise of the term “bioinformatics” and how its linguistic use might reflect trends in the field per se. I will be citing a rather unconventional corpus of editorials, vision statements, government strategy reports, quasi-commercial think tank documents, and the media. This statement is necessary to qualify the approach without alienating readers accustomed to a more academic style. I will examine two key aspects of computational biology, namely, its heavily technological nature and its support role for other biological disciplines [2]. These trends may be useful to anticipate future avenues of research and applications, and explore the fundamental importance of this scientific endeavor for the life sciences [3].

## Declining Trends?

**Figure 1:**
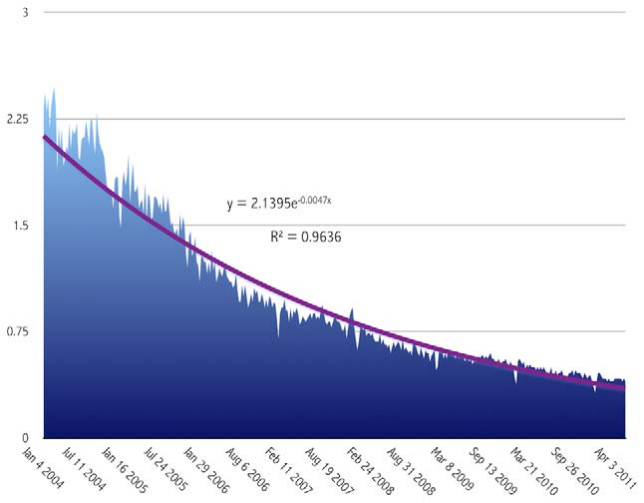
Use of the term “bioinformatics” in Google Trends. The use of the term “bioinformatics” in Google Trends (http://www.google.com/trends?q=bioinformatics&ctab=0&geo=all&date=all&sort=0) plotted with relative scaling, i.e., scaled to the average search traffic for the term (*y*-axis) during the time period (*x*-axis) (for additional explanations, check the About document http://www.google.com/intl/en/trends/about.html). The trend equation and the R^2^ factor are also shown.

One might well wonder whether the term “bioinformatics” is no longer in vogue, compared to those years a decade ago when its use seemed to be associated with great excitement and the anticipation of a new era. A casual look into Google Trends suggests a remarkable pattern of decline in appearances in Google News. To wit, the use of the term “bioinformatics”, largely reflecting news feeds for the discipline, has diminished by almost 6-fold over the past 7 years (Figure 1). The trend equation is an exponential of this form: y = 2.1395e^−0.0047x^ and a R^2^ factor = 0.9636, signifying that the trend may reach y = 0.1, i.e., virtually irrelevance, in x = 651 weeks, or just over a dozen years from now. Such a trend cries out for an explanation. Why is it that a field that appeared unstoppable in all its glory just a few years ago might already be exhibiting signs of (media) fatigue? And does this trend indicate lack of progress, lack of interest, both, or none of the above? We take this graph as a stepping stone, an opportunity to discuss the above questions, bearing in mind that this is a trends analysis and not a strictly scientific discourse on the subject.

## One Explanation: Too Much Promise?

One way to assess the development of bioinformatics and its promised progress is by examining predictions made when the field first entered the limelight. While one might think the field was overly hyped, in fact most past statements have been reasonably balanced, measured, and only subtly evangelical for the establishment of computational research within the life sciences, a monumental task probably accomplished successfully. The selected corpus here covers 15 years or so, split artificially into three periods, which I will define as the “infancy” (1996–2001), “adolescence” (2002–2006), and “adulthood” (2007–2011) periods. This selection was based solely on relevance regarding challenges and opportunities for the field, and does not depend on impact, e.g., status of journal or number of received citations. In this manner, my hope is that this eclectic mix of references is as inclusive as possible, thus better representing a range of opinions voiced during these periods and not too biased by particular specialties, institutions, or journals.

## The “Infancy” Period: 1996–2001

During the “infancy” period, the perception for the wider public, including biologists, was that this was a new field. Yet, much had already happened: the basic ideas were in place, some key algorithms were fully developed, and database resources were being built up [4]. Already, there were debates about the interoperability of database systems with the newly arrived HTTP protocols and other mechanisms, as well as social elements, including international coordination of resources and training requirements [5]. The job market was virtually exploding and demand was exceeding supply: there was a sense that Europe was lagging behind the United States and efforts were put in place to secure funding [6]. This is the time when most graduate programs in bioinformatics were being established, under the guidance of the recently founded International Society for Computational Biology [7]. In a period that feels so long ago, there was a realization that bioinformatics, properly coupled with high-throughput biology, had the potential to transform biomedical research [7], [8]. The terms “flood” and “explosion” as applied to ever-increasing data volumes were in wide use [9] (much more than terms such as “tsunami” or “avalanche”, for some reason); this explosion of sorts was expected to challenge “data organization, accessibility and, most importantly, interpretation” [9]. Many of these challenges remain with us today, in the same order (interpretation being the hardest part). Predictions of “laboratory miniaturization and non-destructive technologies” were heralding the dawn of “systems biology” [10]. On the systems side, it was envisioned that humans would flock to computer systems (not the other way around, as we are experiencing today, in the era of ubiquitous computing) [10]. On the data side, it was noted that the inevitable use of automated approaches had “led to much database misinformation” [11]. This was the era of ontology and vocabulary designs and more extensive database cross-referencing. The nature of the data was “global”: it was genes, sequences, structures, expression profiles, and genomes, reflected in the content of the most well-known molecular biology databases, providing opportunities for the coupling of high-throughput experimentation to computational research [12], [13]. Slowly, the medical fields were embracing high-throughput methodologies and the genomics revolution [14]. In industry, numerous business opportunities existed and the growth outlook was very positive [15]. Away from applications, there was also a conceptual shift in biology, and the opinion that we were moving into a new science, where we would be striving “to develop higher-order algorithms for linking data, structures, and functions in networks” [16]. As a result, funding was increasing: for example, the European Bioinformatics Institute's budget doubled during this period [17]. Infrastructures were already deemed essential for the field to move forward [18]. Issues of data release and accessibility policies [19] as well as intellectual property protected by patents were also emerging during this early period: “the legal treatment of inventions in bioinformatics is in its infancy” [20]. This short journey into the not-so-distant past hopefully provides a flavor of the fluidity of the field during its early period [21]. On the whole, I would suggest that most public statements during this early phase were reasonably measured and did not oversimplify the challenges and anticipated directions of computational biology into the 21st century.

## The “Adolescence” Period: 2002–2006

By now, the field was already in the limelight, after the famous Clinton-Blair handshake for the completion of the human genome in 2000. It made sensational headlines such as “the laboratory rat is giving way to the computer mouse”, partly to explain the multibillion dollar markets [22]. Various agencies were now scrutinizing strategies for the support of the field and playing out different scenarios, for example whether there would be a “Europe-wide integration or coherent strategy” by 2006 [23]. One crucial observation was that bioinformatics was moving outside its comfort zone into new territories with new data types, “toward ‘real’ biology” [24]—this point will be discussed below. Clearly, the impact of expression profiling was being felt in the community [25]. From its humble roots in molecular biology, computational biology was coming up in the world, reaching the realms of computational cell biology [26]. Computation was not only going deeper into the cell, it was becoming broader, too: already, dozens of genomes increasing to 100 or more were being sequenced and, among other issues, multiple genome comparison was now emerging as a topic of research [27]. Structural genomics was being established and faced new challenges, e.g., metadata tracking [28]. Vision statements about the future of biological research were now taking into account the multidisciplinary nature of the field, broadening its horizons [29]. There was now a solid acknowledgment of the human element in the automation utopia previously offered by bioinformatics [30], called the “people paradox”: the realization that “the application of computer science to biology results in an increase in the demand for people” [31]. At the same time, the notion of “personalized medicine” and data sharing in pharmacogenomics [32] increased the stakes and established the flagship role of bioinformatics in this new era [33]. This impact was felt in emerging fields as well, synthetic biology being the latest arrival [34]. Yet, in virtually all expositions, the issue of data integration was repeatedly appearing [35] and was being addressed by the rapid development of bio-ontologies and controlled vocabularies [36]. It seemed that no matter how much effort was in place, the “people paradox” was reemerging to haunt us [37]. This was the time of the appearance of specialized disciplines within the field, e.g., for agriculture [38], generating even more complex and domain-specific data types [39], [40]. Robotics and automation platforms were propagating into medicine rapidly [41], [42]. It was becoming clearer that the fusion of disciplines was far deeper than simply computing and biology [43]: moving into public health, ethical, legal, and social issues needed to be taken into account [44], along with educational or epistemological elements [45]. There were concerns, however, that the pace of discovery and wider applications in medical biotechnology were not delivering against high expectations, with the realization that the otherwise productive “shift from craft-based to more industrialized experimentation” encountered bottlenecks downstream in the discovery process [46]. One factor in policymakers' high expectations might have been a certain lack of milestones: due to the field's dual nature, that of science and engineering, computational biology rarely has the “eureka” moment of a scientist's discovery and is grounded in the laborious yet inspired process of an engineer's invention. At the same time, much effort was being invested in formulating training and curriculum development [47], [48]. We thus reached a turning point, with bioinformatics and computational biology finding its place as a key discipline both within life science and biological technology [49].

## The “Adulthood” Period: 2007–2011

Admittedly, there is no clear dividing line for the next transition. Placing it between years 2006 and 2007 might reflect a certain symmetry—or, on a more personal note, the beginning of a new journey after a long appointment. Nevertheless, it is evident that during the past 5 years, we have moved into a new phase, that—if understood properly—can help us define our future strategy. By 2007, things had become more sophisticated: text mining could now be used in trends analysis of the field for decision making [50], ontology development was proliferating into every aspect of computing [51], and bioinformatics was pervasive in the life sciences, for example, extending to biodiversity conservation planning [52] or synthetic biology [53]. Besides the more theoretical aspects of network biology [54], exemplified by gene and protein interaction networks, pressure mounted for support of translational medicine, ranging from structural variation [55] to cancer bioinformatics [56]. Due to the initial excitement, some mistakes of the past were reappearing, for instance in the reporting of structural variants, for which “there has been no standard approach to collecting the data, assessing its quality or describing identified features” [55]—reminiscent of function annotation a decade earlier. On another level, the challenges were not dissimilar from the ones that the field had been experiencing all along: “managing a huge data volume, integrating information from various discovery platforms and discerning phenotypic implications” [55]. In the midst of this next wave, biologists had to adapt yet again to a bewildering new array of software suites with more emphasis on “user-friendly” software: “biological intuitiveness and investigator empowerment need to take precedence over the current supposition that biologists should re-tool and become programmers when analyzing genome scale datasets” [57]. A “deja-vu” feeling around education and training appeared, for instance with regard to training clinicians in the translational realm of genomic medicine, evidently including bioinformatics [58]. At the same time, new problems were emerging, related to next-generation sequencing efforts, ranging from resequencing to metagenomics [59]. This new data stream was necessarily closer to the platforms generating it, rather than the more detached, “classical” bioinformatics data types (genes, proteins, networks, genomes); it has now become “real” indeed [24]! More traditional problems are still with us today, such as drug [60] and biomarker [61] discovery, data curation [62], literature mining [63], and workflow development [64]. The prediction in 2008 was that in 10 years, we will possess an adequate infrastructure for biological research [65], in a fusion of disciplines [66]. Switching to the present, we are now faced with an expansion of problems, ranging from genome assembly [67], protein design [68], or metagenomics [69] to genomic medicine [70], infectious disease [71], and phenotyping [72]. The latter few deserve verbatim citations, since these activities are also now becoming “real”, very real. On the metagenomics front, it has been noted that “to understand how the Earth breathes, grows, evolves, renews and sustains life is the great adventure now beckoning to us” [69]. For genomic medicine, we hear that “systems medicine should be developed through an international network […] dedicated to inter-disciplinary training and education, to help reduce the gap in healthcare between developed and developing countries” [70] and that as “microorganisms do not follow national borders, such initiatives are probably best started from intergovernmental organizations […], to facilitate the spread of new concepts and software […]” [71]. In a sense, the genomics-bioinformatics nexus has now spilled into the real world [73]. Challenges for health, food and feed, materials, fuels, energy sources. and the environment are all on the agenda [74]. The expectations are high and the stakes have never been greater.

## Another Explanation: Too Much Progress

It might be readily obvious by now in this essay that the “decline” of media interest and the potential diminution of the linguistic use of “bioinformatics” might not reflect the knee-jerk explanation of “too much promise”. As we have suggested above, expectations in the past 15 years have generally been modest and realistic within the community of computational biology. Despite the great challenges of managing outside expectations, commercial opportunity, legal and ethical issues, educational and training needs, as well as multiple disruptive technologies, from the Web to mobile devices, the field has not only contributed to the omics revolution, but also has established a basis for a transformation of biology into a quantitative science. In that sense, an alternative, perhaps fairer, explanation for this apparent decline might be that, indeed, there has been too much progress, if anything. To catalog some of the recent efforts, in an ad hoc manner, one can mention links to synthetic biology [75], [76], protein docking [77], systems medicine or physiology [78], [79], translational [80] and personalized medicine [81], or genome-wide association studies [82]. Despite some negative press coverage at times [83], there has been tremendous progress towards the establishment of computing in virtually every realm of life sciences. Yet, old problems remain with us and should not be neglected, for instance database searches, multiple alignment, orthology detection, structure-function or species relationships, and protein annotation [84]. With a whole new level in data volumes, all these problems come back with a vengeance—including training, once again [85].

## From Global to Local—And Back

The above exposition is an attempt to provide a quick tour of what has happened in the past 15 years in the field. The focus has been the perception of the field and not just the substance: when this investigation began, I was searching for blatant over-statements in the literature, yet I found few. Thus, it can be argued that the declining trend might be attributed mostly to the nature of the field, which found itself in the midst of the turmoil of a wider transformation, driven by industrial and social needs. In other words, it is not lack of interest and definitely not lack of progress: instead, it might be exactly the opposite. The vast progress and the dislocation of traditional biological research into a more precise and quantitative science has moved computational biology from the fringes to the eye of the storm.

Two remarks that address some of the other questions raised above follow. First, the shift from academic exploration to real-world applications and the extension of range both deeply into the cell and widely across all levels of biological organization drives computation to become increasingly local. A few years back, it would be inconceivable that one could remain competitive with “chunks” of data and a workstation. It seemed as if we would be needing more and more storage and compute capacity to execute any significant research and that scaling up was the only way forward. Yet, and thanks to the infrastructures now in place as well as the idiosyncrasies of the new breed of data generation platforms, it is becoming possible to scale down and still explore certain problems effectively. In a subtle yet significant manner, both biological data and computer power seem to be appearing out of the mists of the Internet cloud. Data might be richer, and in small, potent doses and high concentrations can generate fascinating results. Similarly, compute power is becoming available in various ways and unexpected locations. The nature of the game is changing: from an effort to concentrate as much data as possible and throw them to large computers, we might be experiencing something much more effervescent instead. It will be the right mix of data and machines that will derive small packages of high-value products, exciting new science. Biological computation might start producing the equivalent of perfume or spice for scientific and medical research, without excluding of course the bulk, staple food equivalent, which we were accustomed to, until the recent past.

Second, it is interesting that many key questions that have been considered solved are coming back to us on a whole new and different level. We listed some of scientific questions above; these can be coupled to ever present social themes such as the blissful anarchy and subsequent management of novel exciting data types, the cybertopia of automatic data interpretation, the apparently endless need for education and training, the chimera of data integration, and most importantly, the dual nature of the field. An honest self-assessment and the definition of relevant milestones have the potential for supporting the proper public understanding of bioinformatics and better, wiser expectations management.

## Epilogue

The notion of computing in biology, virtually a religious argument just 10 years ago [2], is now enthroned as the pillar of new biology. This is the reason that despite the apparent fatigue, infrastructures for the computational analysis of biological systems are expanding, and moving from research labs into the mainstream. At the same time, this fusion of computational biology with most biology makes it harder for the field to stand out and clearly remain isolated: as predicted years ago, “this discipline will continue to evolve rapidly into the 21st century, perhaps to a point beyond recognition” [4]. It might be that a new, “theoretical biology” is emerging, where models and their predictions can now be assessed by experimental biology, in analogy to the interplay between theoretical and experimental physics. This moment might have come faster than expected. The merging of computation into the fabric of biosciences and biomedicine by 2020, while acquiring a key, critical position amongst other technologies in the toolkit, will possibly necessitate a redefinition of computational biology as a distinct discipline in the not-so-distant future.

**Author's Biography**

Christos A. Ouzounis is a Principal Investigator at the Centre for Research & Technology Hellas (CERTH), Thessalonica, Greece, and a visiting Professor at The Terrence Donnelly Centre for Cellular & Biomolecular Research (CCBR), University of Toronto, Canada. He received his PhD from the University of York, United Kingdom, for research carried out at EMBL (-1993), and was a Human Frontiers Science Program (HFSP) postdoctoral fellow at the AI Center, SRI International in Menlo Park, CA (-1996). He later led the Computational Genomics Group at EMBL's European Bioinformatics Institute (Cambridge, United Kingdom) (-2005), the Computational Genomics Unit at CERTH (-2007), and the Centre for Bioinformatics at King's College London (-2010). He is an Associate Editor for *PLoS Computational Biology*, *PLoS ONE*, and *BioSystems*, has been an Associate Editor for *Bioinformatics*, and an editorial board member of a number of journals and the Faculty of 1000. He is a founding officer of the International Society for Computational Biology (ISCB), the Mikrobiokosmos initiative (Greece), and the Hellenic Society for Computational Biology and Bioinformatics (HSCBB). His scientific interests revolve around genome structure, function and evolution, biological sequence comparison, knowledge representation for genomics, synthetic biology, exobiology, personalized biomedicine, and science communication. He has published over 170 scientific reports, which received over 9,500 citations over 20 years, with an h-index of 54. Some of his best known contributions in the field of computational genomics include automated sequence annotation, the discovery of genomic context methods, the inference of metabolic pathways from genome sequences, the development of methods for large-scale clustering of sequence similarities, the definition of the Last Universal Common Ancestor (LUCA), and the quantification of horizontal gene transfer patterns across the “net of life”. He also maintains a strong interest in the development of computational biology as an exemplary paradigm in the history of contemporary science.
